# The Core Jasmonic Acid-Signalling Module *CoCOI1/CoJAZ1/CoMYC2* Are Involved in Jas Mediated Growth of the Pollen Tube in *Camellia oleifera*

**DOI:** 10.3390/cimb44110366

**Published:** 2022-11-02

**Authors:** Yiyao Liu, Junqin Zhou, Mengqi Lu, Jin Yang, Xiaofeng Tan

**Affiliations:** Key Laboratory of Cultivation and Protection for Non-Wood Forest Trees, Ministry of Education, Central South University of Forestry and Technology, Changsha 410001, China

**Keywords:** *Camellia* oil tree, self-incompatibility, jasmonic acid, expression

## Abstract

*Camellia oleifera* is a woody edible oil species with late self-incompatibility characteristics. Previous transcriptome analysis showed that genes involved in jasmonic acid signal transduction were significantly different in self-and cross-pollinated pistils of *Camellia oleifera*. To investigate the relationship between jasmonate signal and self-incompatibility by studying the core genes of jasmonate signal transduction. The results showed that exogenous JA and MeJA at 1.0 mM significantly inhibited pollen tube germination and pollen tube elongation. and JA up-regulated *CoCOI1*, *CoJAZ1*, and *CoMYC*, the core genes of jasmonate signal transduction. Subcellular localization indicated that CoCOI1 and CoJAZ1 were located in the nucleus and CoMYC2 in the endoplasmic reticulum. The three genes exhibited tissue-specific expression pattern. *CoCOI1* was significantly expressed in pollen, *CoJAZ1* was significantly expressed in ovary, *CoMYC2* was significantly expressed in filaments, but not in pollen. Furthermore, *CoJAZ1* and *CoMYC2* were highly expressing at 24 h in self-pollinated styles. These results suggested that JA signal transduction of *C. oleifera* was involved in the process of self-pollination, and thus in the process of plant defense. When pollen tubes grew slowly in the style, ovary may receive JA signal, which initiates the molecular mechanism of inhibiting the growth of self-pollinating pollen tubes.

## 1. Introduction

The Theaceae *camellia* family member *Camellia oleifera* is a significant species of woody edible oil tree in southern China. The “Oriental olive oil” is a high nutritional content, high quality woody edible vegetable oil that is produced by one of the world’s four major woody oil plants, along with the olive, oil palm, and coconut [1,2]. Squalene, tocopherol, vitamins, carotene, triterpene alcohol, saponins, and other active components are abundant in oil [3]. China has a long history of *C. oleifera* cultivation and is the world’s largest producer of seed oil. For a long time, the phenomenon of more flowers and fewer fruits has existed, and its self-fertility is low under natural conditions, owing to genetic characteristics such as self-incompatibility and improper pollination variety arrangement [4]. *Camellia oleifera* is a late-acting self-incompatibility (LSI) tree species, understanding the mechanism may improve seed yield [5].

The LSI response is speculated to be divided into two stages: the first stage slows down the growth rate of the pollen tube in the style. In the second stage, pollen tube growth is inhibited in the ovary, which is preparing to reject self-pollen tubes [6]. Self-incongruence comes from an old plant protection framework, it can influence dust acknowledgment and direct dust tube development [7,8]. Plants have evolved complex defense systems and established a complete set of signal transduction systems, which play a core role in the potential stress signal transduction network [9]. Jasmonic acid (JA) and ethylene (ET) are two major plant hormones involved in regulating plant defense responses, widely existing in plants. Exogenous stimulation can stimulate the expression of plant genes and induce plant defense [10,11]. When stimulated by external stimuli, plants respond through signal networks, JA and ET produce responses, JA acts on *ERF1* and *MYC2* genes through *COI1*, *ET* directly acts on *ERF1* genes, and finally activate defense response genes [12].

Zhou et al. [6] found that when the self-pollinating pollen tube of *C. oleifera* grew from the base of style to the upper part of ovary, abnormal phenomena such as swelling, bifurcating and wave appeared, and then stopped growing. The results confirmed that belongs to LSI of Programmed cell death (PCD) type. PCD is a genetically determined active suicide process that plays an important role in plants under biological and abiotic stress [13,14]. Wang et al. [15] proved that PCD plays a key role in pears and that actin depolymerization activity of PbrS-RNase plays an important role in promoting PCD in pear pollen tubes. The mechanism of self-incompatibility is also regulated by PCD of self-flowering pollen in *Papaver somniferum* L. [16].

Jasmonic acid (JA), methyl jasmonate (MeJA) and JA isoleucine (JA-Ile) are collectively referred to as Jasmonates (JAs) [17]. Yamane et al. [18] showed that JA could inhibit pollen germination of *Camellia sinensis*, but MeJA could not. Spraying of exogenous methyl jasmonate can significantly reduce the pollen germination rate of *Brassica napus* L., and affect the flowering time and formation of flower organs [19]. Zeng et al. [20] studied that DHJA and MeJA significantly inhibited rice pollen germination, and when the concentration was 2.5 mM, it almost completely inhibited pollen germination, and pollen burst and spilled. JAs may act as an endogenous pollen germination regulator to inhibit pollen germination.

JAs was initially thought to be a stress-related hormone, which is also involved in the regulation of important growth and development processes [21]. They play an important role in regulating plant growth and development, pollen germination, flower opening, pistil development and other aspects [19,20,22,23]. When plants are stimulated by external environment, that is, when jasmonic acid level increases, a large amount of jasmonic acid is synthesized and JA-Ile is formed under the action of ATP-dependent jasmonic acid resistant 1 (JAR1), which promotes the binding of JAZ protein and SCF^COI^ complex. The SCF^COI^-JA-ILE complex is formed to ubiquitinated JAZs protein and degraded by the 26S proteasome pathway, resulting in the separation of MYC2-JAZ-NINJA-TPL complex and the release of inhibition of transcription factor MYC2, thereby activating the transcription of jasmonic acid responsive genes [24,25,26]. In 1998, Xie et al. [27] cloned *COI1* (*COR*-*insensitive 1*), which opened the prelude of jasmonine signal transduction research. As the only receptor of jasmonic acid signal transduction pathway, COI1 plays a very core role in jasmonic acid signal transduction. The transcription suppressor JAZ (Jasmonate zim-domain) is the direct substrate of the SCF^COI^ ubiquitin ligase complex in JA signaling, JAZs members contain an intermediate ZIM conserved domain and a Jas conserved domain near the C-terminal [28]. The SCF^COI^ complex binds to JAZ1 protein in the Jas domain and mediates jasmonic acid signaling pathway to positively regulate the inhibition of transcription factor MYC2. MYC (Myelocytomatosis proteins), as transcriptional activators of jasmonic acid signaling pathway, also play an important role in signal transduction [29]. Among the discovered MYC transcription factors, MYC2 is the most thoroughly studied one. MYC2 is member of the Basic helix-loophelix (bHLH) family and interact with most JAZs [30].

This study shows that the jasmonic acid signal transduction pathway is involved in the mechanism of C. oleifera self-incompatibility response, The relationship between the core Jasmonic acid-signalling Module and LSI, and lays a foundation for analyzing, the molecular mechanism of *C. oleifera* self-incompatibility.

## 2. Materials and Methods

### 2.1. Plant Materials, Pollination Treatment, and Sample Collection

Five-year-old *C. oleifera* varieties, “Huashuo” and “Huajin” growing in Dongcheng Town, Wangcheng District, Changsha City, Hunan Province, China. Two pollination treatments were designed, including “Huashuo” × “Huashuo” (self-pollination) and “Huashuo” × “Huajin” (cross-pollination). Pollination and sampling took place on sunny days in Oct. 2018, as QRT-PCR sample. At 0, 2, 12, 24, 36, 48, 60, 72, 84 h after self-cross pollination, the pistils were removed and divided into ovary and style, and the un pollinated “Huashuo” lower bud in the exposed state was taken, and petal (Pe), anther (An), filament (Fi), style (St), pollen (Po) and ovary (Ov) were collected and wrapped in tin foil, frozen immediately in liquid nitrogen, and stored at −80 °C. All groups of samples had three biological replicates.

### 2.2. Pollen Culture

To explore the effects of JA and MeJA on pollen development by cultivation of *Camellia oleifera* pollen with exogenous hormones. “Huashuo” pollen grains was cultured at 28 °C in the germination medium which containig 0.015% H3BO3, 10% sucrose, pH 7.0. Scatter the pollen evenly in the pollen liquid medium of JA (98%, Macklin) and MeJA (95%, Sigma-Aldrich). The JA, MeJA concentration gradients were 0, 0.05, 0.1, 0.25, 0.5 and 1.0 mM, both JA and MeJA solutions were filter sterilized with a filter membrane (0.22 μm, MILLIPORE). Shake gently 2 h of culture, pollen germination was observed under a microscope (Olympus IX71, Japan), the experiment was repeated for three times, each repetition randomly selected 3 visual fields and more than 100 pollen grains. The germination criterion was that the length of the pollen tube was longer than the diameter of the pollen grain, using ImageJ to measure the pollen germination and the length of pollen tube.

### 2.3. Extraction of RNA and Quantitative RT-PCR

Quantitative RT-PCR was used to explore the expression patterns of related genes in self-pollination and cross-pollination of *Camellia oleifera*. Total RNA was extracted using E.Z.N.A Plant RNA Kit (Omega Bio-Tek, Norcross, Georgia), 1.2% agarose gel electrophoresis to judge the integrity and overall quality of total RNA. Reverse transcription of each sample was carried out with HiScript^®^ II Q RT SuperMix (+gDNA wiper) (Vazyme, Nanjing, China). PCRs were performed using ChamQ Universal SYBR qPCR Master Mix (Vazyme, Nanjing, China) on a CFX96 Real-time PCR system (BIO-RAD). Primers were designed for use in quantitative RT-PCR (qRT-PCR).

(CoCOI1: F5′-ATCAAAGAGAAAGATGGAGAATG-3′, R5′-CAGCACTAAAGAAACCAATAAGA-3′; CoJAZ1: F 5′-CCTCAGGCTCGCTAACTCA-3′, R5′-CGATGCCCTTCTTGCTATT-3′; CoMYC2: F 5′-CCCATCAGAGACCGAAAGACT-3′, R5′-ACCAAGGAGCCAGAACTAAAAC-3′). GAPDH was used as an internal control gene for normalization Quantitative variations were calculated by the relative quantification method (2^−ΔΔCT^) and three independent.

### 2.4. Gene Cloning and Bioinformatics Analysis

The RNA was reverse transcribed into cDNA using the HiScript^®^ II 1st Strand cDNA Synthesis Kit (+gDNA wiper) (Vazyme, Nanjing, China). Refer to the instructions of PrimeSTAR^®^ HS (Premix) (VAzyme, Nanjing, China) for PCR amplification. PCR-amplified products of expected sizes were gel-purified using a Gel Extraction Kit (OMEGA, San Francisco, CA, USA), and transformed into a coli DH5α (TIANGEN Biotech, Beijing, China). The physical and chemical properties such as molecular weight, amino acid composition, and theoretical isoelectric point (pI) were predicted by online tool ProtParam (https://web.expasy.org/protparam/, accessed on 1 October 2022). The amino acid sequences of JA family proteins from Cs, *Arabidopsis thaliana*, *Brassica rapa*, *Glycine max*, *Oryza sativa*, *Vitis vinifera* and *Zea mays* were retrieved from NCBI and examined together. Phylogenetic tree of gene was built after alignment of full-length protein sequences using neighbor-joining. Analyses were conducted using MEGA6.

### 2.5. Subcellular Localization

Subcellular localization using the pCambia1300 construct of the coding sequence with a GFP tag driven by a CaMV35S promoter, and the endoplasmic reticulum was labeled with CD3-959-mcherry. All of the fusion constructs were transformed into Agrobacterium tumefaciens strain GV3101, and the bacterial suspension of CD3-959-mCheey (OD600 = 1.0) was infiltrated into fully expanded leaves of 4-week-old *N. benthamiana* leaves with the equal proportion of Agrobacterium (OD600 = 1.0) using needleless ayringe. The fluorescence signals were examined after plants were grown under dark for 6 h and then grown under light for 48 h by a confocal laser scanning microscope (LSM510, LEICA, Wetzlar, Germany) after staining with DAPI in the dark for 10 min. The fluorescence of green (GFP), red (mCherry) and blue (DAPI) fluorescent proteins was excited at 488, 552 and 405 nm, respectively.

### 2.6. Data Analysis

All the data were statistically analyzed using SPSS 22.0 (IBM Corp., Armonk, NY, USA). Multiple comparisons of means were carried out using Duncan’s multiple range tests (DMRT).

## 3. Results

### 3.1. Exogenous JA and MeJA Inhibit Pollen Germination and Tube Growth

**Figure 1:**
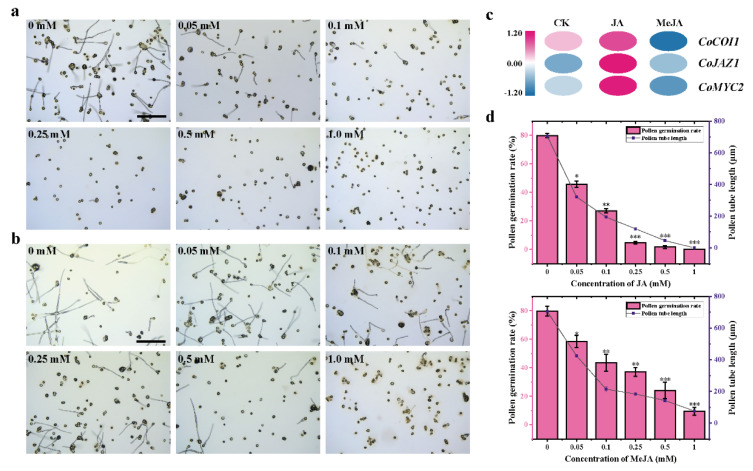
JA and MeJA affect pollen development of *C. oleifera*. (**a**,**b**) Pollen of *C. oleifera* was cultured with different concentrations of JA and MeJA. Bar = 500 μm. (**c**) Expression of key genes about jasmonic acid-signalling module in *C. oleifera* pollen. (**d**) Pollen germination rate and pollen tube length of *C. oleifera*. * *p* < 0.05; ** *p* < 0.01; *** *p* < 0.001.

Exogenous JA and MeJA were added to the pollen liquid medium to treat the pollen. The results showed that exogenous JA and MeJA significantly inhibited the pollen development of *C. oleifera*, and the effect of JA was more significant (Figure 1b). 1.0 mM JA could completely inhibit the pollen germination of *C. oleifera* (Figure 1a,d). The expression of *CoCOI1*, *CoJAZ1* and *CoMYC2* gene in pollen of *C. oleifera* cultured with 0.5 mM JA was increased by qRT-PCR (Figure 1c).

### 3.2. Cloning and Bioinformatics Analysis of CoCOI1/CoJAZ1/CoMYC2

**Figure 2:**
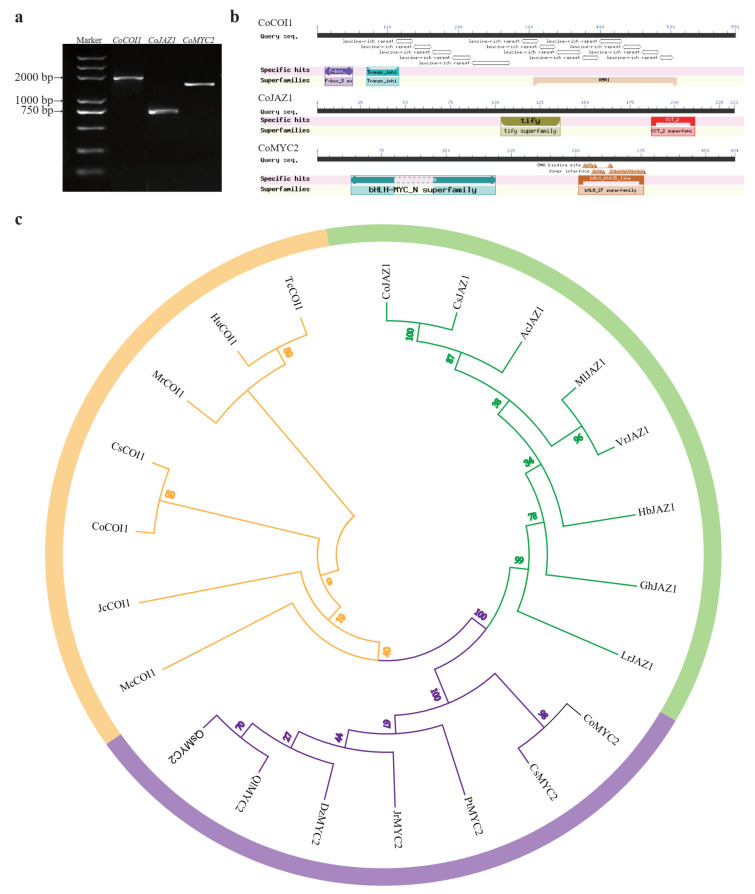
Cloning and bioinformatics analysis of CoCOI1/CoJAZ1/CoMYC2. (**a**) Agarose gel electrophoresis of full-length CDS sequences of *CoCOI1/CoJAZ1/CoMYC2*. (**b**) Domain analysis of CoCOI1/CoJAZ1/CoMYC2. (**c**) Phylogenetic tree of CoCOI1/CoJAZ1/CoMYC2. McCOI1 (XP_022156918.1); ZjCOI1 (XP_015877284.1); TcCOI1 (XP_007009091.2); HuCOI1 (XP_021295823.1); JcCOI1 (XP_012087930.1); MrCOI1 (KAB1217778.1); CsCOI1 (ANB66331.1); MLJAZ1 (AEC12208.1); AtJAZ1 (NP_001319041.1); SmTIFY10A (ATA66294.1); GhJAZ1 (AIV42065.1); AcJAZ1 (PSS18120.1); CsJAZ1 (ANB66339.1); LrJAZ1 (QDA11338.1). PtMYC2 (XP_002301432.1); PeMYC2 (XP_011023113.1); JrMYC2 (XP_035548572.1); QsMYC2 (XP_023880894.1); QlMYC2 (XP_030931929.1); MnMYC2 (XP_010100678.1); DzMYC2 (XP_022769754.1); MrMYC4 (KAB1219718.1); CsMYC2 (XP_028063164.1), Bootstrap is set to 1000 repetitions.

Through the analysis of the transcriptome database of *Camellia oleifera* (Zhou et al., 2020), three differentially expressed genes in the jasmonic acid-signalling were identified. They are named *CoCOI1*, *CoJAZ1* and *CoMYC2,* and their CDS sequences were cloned by RT-PCR (Figure 2a). CoCOI1, CoJAZ1 and CoMYC2 had the highest similarity of more than 95% with CsCOI1 (ANB66331.1), CsJAZ1 (ANB66339.1), and CsMYC2 (XP_028063164.1), and shared more than 70% similarity with other species. Their CDS sequence lengths were 1776, 702, and 1455 bp, encoding 591, 233, and 484 amino acids. The predicted molecular formulas are C_2970_H_4740_N_828_O_859_S_33_, C_2970_H_4740_N_828_O_859_S_33_, and C_2970_H_4740_N_828_O_859_S_33_, relative molecular mass were 66.85, 25.39, and 53.38 kDa.

Through the conservative domain analysis on NCBI online website, the prediction results show that CoCOI1 has a typical F-box (12−51 aa) (pfam18511) conservative domain at the N-terminal and 13 leucine rich repeat LRRs (111−504 aa) domains at the C-terminal, the F-box can be coupled with the SkpⅠ protein of SCF to form the SCFCOI complex, and the LRRs domain at the C-terminal can specifically recognize and bind to the substrate, which is indispensable in jasmonic acid signal transduction. CoJAZ1 contains two highly conserved domains: a typical TIFY domain (103−136 aa) (pfam06200) and a CCT-2 motif (187−211 aa) (pfam09425). CoMYC2 with a typical bHLH-MYC-N (40−207 aa) (pfam14215) conserved domain and HLH domain (40−207 aa) (pfam14215), is a bHLH-type transcription factor (Figure 2b).

In order to study the evolutionary relationship between CoCOI1/CoJAZ1/CoMYC2 and other species, MEGA7.0 was used to construct a phylogeny of CoCOI1, CoJAZ1, CoMYC2 and tea plant related proteins by neighbor-joining (NJ) method. The phylogenetic tree results show that the jasmonic acid-signalling module proteins of *C. oleifera* are most closely related to the tea tree.

### 3.3. Subcellular Localization of CoCOI1/CoJAZ1/CoMYC2

**Figure 3:**
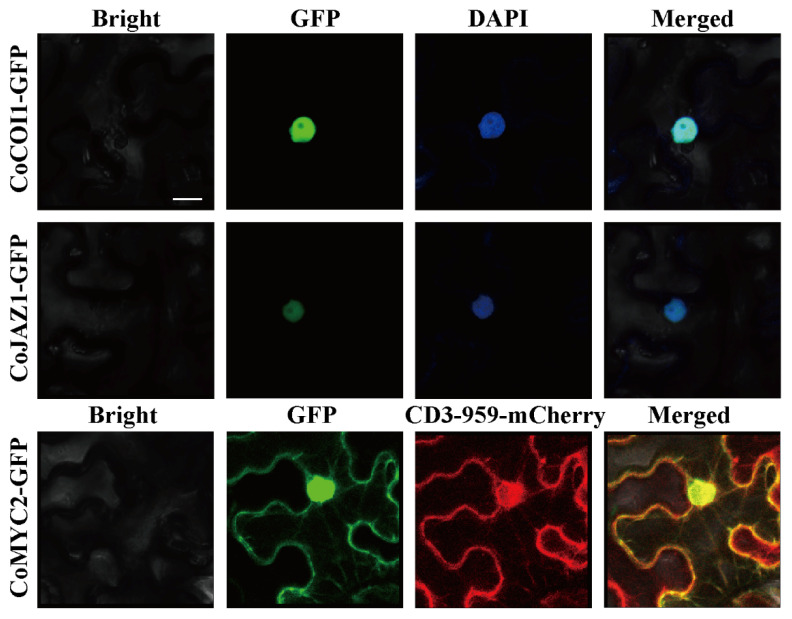
Subcellular localization of CoCOI1/CoJAZ1/CoMYC2 in *Nicotiana benthamiana*. GV3101 containing the mentioned genes were infiltrated in the leaves of *N. benthamiana*, and the expression was detected with a laser confocal microscope 48 h later. Bar = 10 μm.

To explore the subcellular localization of CoCOI1, CoJAZ, and CoMYC2, the CoCOI1-GFP, CoJAZ1-GFP, and CoMYC2-GFP fusion proteins were transiently expressed in the leaves of *N. benthamiana*. The results showed that the green fluorescence of each fusion protein overlapped with the fluorescence of the nucleus stained by DAPI, indicating that CoCOI1 and CoJAZ1 were located in the nucleus; the CoMYC2 fusion protein overlapped with the fluorescence of the endoplasmic reticulum stained by CD3-959-mCherry (Figure 3).

### 3.4. Expression Patterns of CoCOI1/CoJAZ1/CoMYC2

**Figure 4:**
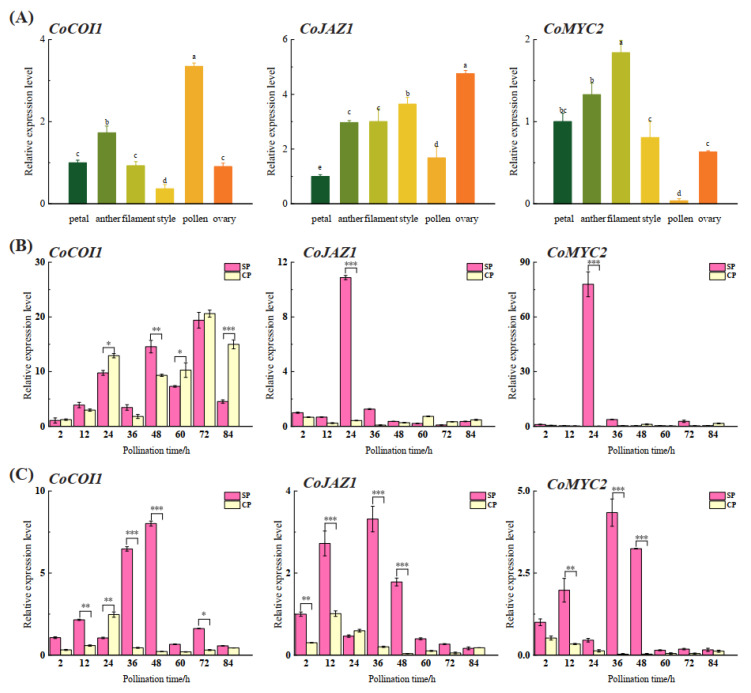
Expression levels of *CoCOI1*, *CoJAZ1*, and *CoMYC2*. (**A**) Expression levels of *CoCOI1*, *CoJAZ1*, and *CoMYC2* in different tissues. The expression levels were measured by qRT-PCR and standardized by *CoGAPDH*. Lowercase letters represent significant differences in the expression levels in various tissues (*p* < 0.05). Error bars show±SD. (**B**) Expression levels of *CoCOI1*, *CoJAZ1*, and *CoMYC2* in self- or cross-pollinated styles. The transcript levels were measured by qRT-PCR and standardized by *CoGAPDH*. The asterisks represent significant differences in expression levels (Student’s *t*-test): * *p* < 0.05; ** *p* < 0.01; *** *p* < 0.001. Error bars show ± SD. (**C**) Expression levels of *CoCOI1*, *CoJAZ1*, and *CoMYC2* in self- or cross-pollinated ovary.

Total RNA was extracted from the mature petal, anther, filament, style, pollen, and ovary of “Huaxin”. The expression levels of *CoCOI1*, *CoJAZ1*, and *CoMYC2* in different tissues were detected by qRT-PCR, and showed great difference (Figure 4a). The results showed that *CoCOI1* was significantly expressed in pollen; *CoJAZ1* was significantly expressed in ovary, *CoMYC2* was significantly expressed in filaments, but not in pollen.

The expression patterns of *CoCOI1*, *CoJAZ1*, and *CoMYC2* in self- or cross-pollinated Style and ovary were also detected by qRT-PCR. In the style the expression levels of *CoJAZ1* and *CoMYC2* were significantly expressed at 24 h in self-pollination, were significantly higher than other gene (Figure 4b). In the ovary, *CoCOI1*, *CoJAZ1*, and *CoMYC2* all showed similar expression patterns after self-pollination, the “double peak curve” expression pattern of “rise decline rise”: the expression of genes increased 2–12 h after self-pollination, and significantly reversed at 24 h after self-pollination, followed by a very significant upward trend. *CoJAZ1* and *CoMYC2* were very significantly expressed at 36 h, and *CoCOI1* was very significantly expressed at 48 h. There was no significant expression after 24 h of cross pollination (Figure 4c).

## 4. Discussion

As a healthy edible oil, with the improvement of life quality, the demand for *Camellia* oil is increasingly, but the yield is not high. The essence of self-incompatibility is the mechanism by which pistil specifically recognizes and rejects pollen with the same genotype [31]. Its late self-incompatibility was one of the important factors restricting its yield [32]. Studies have shown that JAs can inhibit pollen germination of rice [20], loofah and rape [33]. Studies have found that spraying exogenous MeJA could significantly reduce the pollen germination rate of *Brassicanapus L.*, and affect the flowering time and flower organs formation [19]. In tea tree, only JA can inhibit pollen germination, but MeJA cannot [18]. At present, the effect of JAs on pollen germination of *C. oleifera* has not been reported. In view of the homology of *Camellia* oleifera and *Camellia sinensis*, JA and MeJA were used to conduct in vitro germination experiments on pollen. The results showed that both JA and MeJA could inhibit pollen tube germination and elongation, and jasmonic acid had a more obvious inhibitory effect. Pollen germination did not occur at 0.10 mM JA concentration, which was consistent with the results of previous studies, but different from the results in tea plants. In this study, it was found for the first time that JAs could inhibit pollen germination in vitro of *C. oleifera*. In order to preliminatively explore the effects of jasmonic acid substances on the expression of JA signal transduction-related genes, a fluorescence quantitative experiment was conducted. The results showed that module *CoCOI1/CoJAZ1/CoMYC2* were significantly expressed under JA treatment, indicating that exogenous jasmonic acid and endogenous jasmonic acid can stimulate jasmonic acid signal transduction. In the process of flowering and fruiting, hormone level is closely related to pollen quality, flower organ development and fruit setting rate. The pollen tube development of *Camellia oleifera* affect the fruit set rate of *Camellia oleifera*, which has important reference value for variety selection and high-yield cultivation of *Camellia oleifera*.

The late self-incompatibility of *Camellia oleifera* may be due to self-pollination activating the defense mechanism, leading to the programmed death of pollen tubes, resulting in pollination failure and self-incompatibility. Transcriptomic data analysis revealed three JA signal core genes *COI1/JAZ1/MYC2*, were cloned with full-length CDS sequences to obtain gene sequences. CoCOI1 protein has an F-box domain and 13 Leucine-rich repeat LRRs domains. CoJAZ1 contains two highly conserved domains: a typical TIFY domain and a CCT-2 motif. CoMYC2 protein is a bHLH type transcription factor with typical BHLH-MYC-N and HLH domains. Phylogenetic tree analysis showed that the three proteins were most closely related to the related proteins in *Camellia sinensis*, which suggested that they might have similar functions. In order to verify the localization of related proteins in cells, the cloned gene and GFP labeled pCAMBIA1300 vector were used to construct a fusion expression vector using T4 ligase, which was injected into tobacco epidermis mediated by Agrobacterium tumefaciens for transient expression. The results showed that CoCOI1 and CoJAZ1 might be nuclear localization proteins, which was consistent with previous studies, where JAZ protein was nuclear localization signal jas domain, making JAZ a nuclear localization protein [34]. Withers et al. [35] found that MYC2 was expressed in the nucleus and cytoplasm, and CoMYC2 was expressed in the endoplasmic reticulum in this study.

As a receptor for JA transduction pathway, *CoCOI1* is significantly expressed in pollen. Previous studies have shown that SCF complex plays a key role in GSI mediated by S-RNase: SCF can ubiquitinate non-own S-RNase, and *COI1* is a component of SCF complex. The specific expression of *CoCOI1* in pollen may play a key role in self-incompatibility. *CoJAZ1* is specifically expressed in the ovary. According to the accumulation sites of genes under natural conditions, it is speculated that jasmonic acid signal transduction related genes are related to the interaction mechanism of ovary-style signal recognition in *Camellia oleifera.* The expression patterns of module genes in *C. oleifera* at different times after pollination were analyzed. *CoJAZ1* was extremely significantly expressed at 24 h of self-crossing and then showed a downward trend. It as a transcription inhibitor, was released from *CoMYC2* inhibition after 26S ubiquitination [24], and *CoMYC2* was extremely significantly expressed at 24 h of self-crossing. They are not activated under natural conditions, and when the JA level increases, their expression is activated after 26S ubiquitination degradation, indicating that JA signal transduction begins in the style 24 h after pollination, and *MYC2* accumulation occurs. Jasmonic acid (JA) may cause pollen tube PCD by increasing the expression level of S-RNase. In Lycopersicon esculentum Miller, COI1 gene participates in AAL toxin induction and regulation of Programmed cell death (PCD) in tomato cells depending on JA and ET transduction pathways [36].Studies have shown that both endogenous and non-endogenous SRNase entry into the pollen tubes of *Malus domestica* stimulates jasmonic acid production and induces accumulation of *MdMYC2* transcripts, which act as transcription factors in JA signaling pathway and participate in plant defense processes [37]. This is consistent with the results of this study. Interestingly, JA signal transduction core genes showed different in styles after pollination, but similar expression pattern in ovary: a “bimodal curve” expression pattern after self-cross-pollination. It suggests that jasmonic acid responds to regulation in the ovary after self-pollination, and signals may interact in the style and ovary after self-pollination. When the pollen tube is growing, the ovary receives signals to prepare for inhibiting the growth of the self-pollinating pollen tube, which is the second stage of LSI [38], consistent with previous speculation. Among them, *CoJAZ1* showed a similar or even higher expression at 12 h than at 36 h, which may be due to its characteristics as a transcription inhibitor in the initial response to regulation. As an F-box domain protein, *CoCOI1* can form a complex with SCF and participate in JA-regulated plant defense response to biological stress. It may selectively degrade non-self SRNase in SI reaction through protease degradation system, thus causing pollen tube PCD [39,40].

## 5. Conclusions

It was found that the core jasmonic acid-signalling module *CoCOI1/CoJAZ1/CoMYC2* were involved in the reaction mechanism of self-incompatibility of *Camellia oleifera*. During the slow growth of the pollen tube after self-pollination, the ovary may be preparing to inhibit the growth of self-pollinating pollen tube, but the specific molecular mechanism needs further study.

## Data Availability

Not applicable.

## References

[1] Zhuang R.L. (2008). Oil-tea Camellia in China.

[2] Zhang D.L., Stack L., Zhang R.Q., Yu J.F., Xie B.X., Chen Y.Z., Ruter J.M. (2006). Teaoil *Camellia*-Eastern “Olive” for the World. Acta Hortic..

[3] Xiao Y.G. (2006). Investigation and prospect of bio-active components in vegetable oil. Cereal Food Ind..

[4] Li Z.J., Zeng Y.R., Dai W.S. (2009). Studies on internal and external factors which related with low yield and poor benefit in *Camellia oleifera*. J. For. Eng..

[5] Chao G., Yuan D.Y., Yang Y., Wang B.F., Liu D.M., Zou F., Tan X.F. (2015). Anatomical characteristics of self-incompatibility in *Camellia oleifera*. Sci. Silvae Sin..

[6] Zhou J.Q., Lu M.Q., Yu S.S., Liu Y.Y., Yang J., Tan X.F. (2020). In-depth understanding of *Camellia oleifera* self-incompatibility by comparative transcriptome, proteome and metabolome. Int. J. Mol. Sci..

[7] Schopfer C.R., Nasrallah M.E., Nasrallah J.B. (1999). The male determinant of self-incompatibility in Brassica. Science.

[8] Kitashiba H., Nasrallah J.B. (2014). Self-incompatibility in Brassicaceae crops: Lessons for interspecific incompatibility. Breed. Sci..

[9] Campos M.L., de Almeida M., Rossi M.L., Martinelli A.P., Litholdo Junior C.G., Figueira A., Rampelotti-Ferreira F.T., Vendramim J.D., Benedito V.A., Peres L.E. (2009). Brassinosteroids interact negatively with jasmonates in the formation of anti-herbivory traits in tomato. J. Exp. Bot..

[10] Anderson J.P., Badruzsaufari E., Schenk P.M., Manners J.M., Desmond O.J., Ehlert C., Maclean D.J., Ebert P.R., Kazan K. (2004). Antagonistic interaction between abscisic acid and jasmonate-ethylene signaling pathways modulates defense gene expression and disease resistance in *Arabidopsis*. Plant Cell..

[11] Melotto M., Mecey C., Niu Y., Chung H.S., Katsir L., Yao J., Zeng W., Thines B., Staswick P., Browse J. (2008). A critical role of two positively charged amino acids in the Jas motif of *Arabidopsis* JAZ proteins in mediating coronatine- and jasmonoyl isoleucine-dependent interactions with the COI1 F-box protein. Plant J..

[12] Lorenzo O., Chico J.M., Sánchez-Serrano J.J., Solano R. (2004). JASMONATE-INSENSITIVE1 encodes a MYC transcription factor essential to discriminate between different jasmonate-regulated defense responses in *Arabidopsis*. Plant Cell.

[13] Orzaez D., de Jong A.J., Woltering E.J. (2001). A tomato homologue of the human protein PIRIN is induced during programmed cell death. Plant Mol. Biol..

[14] Ma W.W., Xu W.Z., Xu H., Chen Y.S., He Z.Y., Ma M. (2010). Nitric oxide modulates cadmium influx during cadmium-induced programmed cell death in tobacco BY-2 cells. Planta.

[15] Wang C.L., Xu G.H., Jiang X.T., Chen G., Wu J., Wu H.Q., Zhang S.L. (2009). S-RNase triggers mitochondrial alteration and DNA degradation in the incompatible pollen tube of *Pyrus pyrifolia* in vitro. Plant J..

[16] Thomas S.G., Franklin-Tong V.E. (2004). Self-incompatibility triggers programmed cell death in *Papaver* pollen. Nature.

[17] Ruan J.J., Zhou Y.X., Zhou M.L., Yan J., Khurshid M., Weng W.F., Cheng J.P., Zhang K.X. (2019). Jasmonic Acid Signaling Pathway in Plants. Int. J. Mol. Sci..

[18] Yamane H., Takagi H., Abe H., Yokota T., Takahashi N. (1981). Identification of Jasmonic acid in three species of higher plants and its biological activities. Plant Cell Physiol..

[19] Pak H., Guo Y., Chen M.X., Chen K.M., Li Y.L., Hua S.J., Shamsi I., Meng H.B., Shi C.G., Jiang L.X. (2009). The effect of exogenous methyl jasmonate on the flowering time, floral organ morphology, and transcript levels of a group of genes implicated in the development of oilseed rape flowers (*Brassica napus* L.). Planta.

[20] Zeng X.C., Jiang H.Y., Wu X.Y., Fang J.H., Xu F.F. (2003). Effects of jasmonates on pollen Germination in rice. Acta. Agric. Univ. Jiangxiensis..

[21] Campos M.L., Kang J.H., Howe G.A. (2014). Jasmonate-triggered plant immunity. J. Chem. Ecol..

[22] Feng M.J., Xu H., Zhang H., Zhu Y. (2015). Recent progress in jasmonates regulation of plant growth and development. Plant Physiol. J..

[23] Li L., Zhao Y.F., McCaig B.C., Wingerd B.A., Wang J.H., Whalon M.E., Pichersky E., Howe G.A. (2004). The tomato homolog of CORONATINEINSENSITIVE1 is required for the maternal control of seed maturation, jasmonate-signaled defense responses, and glandular trichome development. Plant Cell.

[24] Chini A., Fonseca S., Fernández G., Adie B., Chico J.M., Lorenzo O., García-Casado G., López-Vidriero I., Lozano F.M., Ponce M.R. (2007). The JAZ family of repressors is the missing link in jasmonate signalling. Nature.

[25] Katsir L., Chung H.S., Koo A.J., Howe G.A. (2008). Jasmonate signaling: A conserved mechanism of hormone sensing. Curr. Opin. Plant Biol..

[26] Thines B., Katsir L., Melotto M., Niu Y., Mandaokar A., Liu G., Nomura K., He S.Y., Howe G.A., Browse J. (2007). JAZ repressor proteins are targets of the SCF^COI1^ complex during jasmonate signalling. Nature.

[27] Xie D.X., Feys B.F., James S., Nieto-Rostro M., Turner J.G. (1998). COI1: An Arabidopsis gene required for jasmonate-regulated defense and fertility. Science.

[28] Pauwels L., Barbero G.F., Geerinck J., Tilleman S., Grunewald W., Pérez A.C., Chico J.M., Bossche R.V., Sewell J., Gil E. (2010). NINJA connects the co-repressor TOPLESS to jasmonate signalling. Nature.

[29] Boter M., Ruíz-Rivero O., Abdeen A., Prat S. (2004). Conserved MYC transcription factors play a key role in jasmonate signaling both in tomato and *Arabidopsis*. Genes Dev..

[30] Cheng Z.W., Sun L., Qi T.C., Zhang B.S., Peng W., Liu Y.L., Xie D.X. (2011). The bHLH transcription factor MYC3 interacts with the jasmonate ZIM-domain proteins to mediate jasmonate response in *Arabidopsis*. Mol. Plant..

[31] Goldberg E.E., Kohn J.R., Lande R., Robertson K.A., Smith S.A., Igić B. (2010). Species selection maintains self-incompatibility. Science.

[32] Liao T., Yuan D.Y., Zou F., Gao C., Yang Y., Zhang L., Tan X.F. (2014). Self-sterility in *Camellia oleifera* may be due to the prezygotic late-acting self-incompatibility. PLoS ONE.

[33] Feys B., Benedetti C.E., Penfold C.N., Turner J.G. (1994). *Arabidopsis* Mutants Selected for Resistance to the Phytotoxin Coronatine Are Male Sterile, Insensitive to Methyl Jasmonate, and Resistant to a Bacterial Pathogen. Plant Cell.

[34] Grunewald W., Vanholme B., Pauwels L., Plovie E., Inzé D., Gheysen G., Goossens A. (2009). Expression of the *Arabidopsis* jasmonate signalling repressor JAZ1/TIFY10A is stimulated by auxin. EMBO Rep..

[35] Withers J., Yao J., Mecey C., Howe G.A., Melotto M., He S.Y. (2012). Transcription factor-dependent nuclear localization of a transcriptional repressor in jasmonate hormone signaling. Proc. Natl. Acad. Sci. USA.

[36] Zhang L.P., Jia C.G., Liu L.H., Zhang Z.M., Li C.Y., Wang Q.M. (2011). The involvement of jasmonates and ethylene in *Alternaria alternata f. sp. lycopersici* toxin-induced tomato cell death. J. Exp. Bot..

[37] Gu Z.Y., Li W., Doughty J., Meng D., Yang Q., Yuan H., Li Y., Chen Q.J., Yu J., Liu C.S. (2019). A gamma-thionin protein from apple, MdD1, is required for defense against S-RNase-induced inhibition of pollen tube prior to self/non-self recognition. Plant Biotechnol. J..

[38] Yu S.S., Zhou J.Q., Lu M.Q., Liu Y.Y., Yang J. (2019). Cloning and expression analysis of *Camellia oleifera* transporter gene *ABCB26*. Plant Physiol. J..

[39] Matsumoto D., Yamane H., Abe K., Tao R. (2012). Identification of a Skp1-like protein interacting with SFB, the pollen S determinant of the gametophytic self-incompatibility in *Prunus*. Plant Physiol..

[40] Xu C., Li M.F., Wu J.K., Guo H., Li Q., Zhang Y., Chai J.J., Li T.Z., Xue Y.B. (2013). Identification of a canonical SCF^SLF^ complex involved in S-RNase-based self-incompatibility of *Pyrus* (Rosaceae). Plant Mol. Biol..

